# Feature Reviews in Cardiovascular Diseases

**DOI:** 10.3390/biomedicines13112580

**Published:** 2025-10-22

**Authors:** Arturo Cesaro, Serafino Fazio

**Affiliations:** 1 Department of Translational Medical Sciences, University of Campania “Luigi Vanvitelli”, 80131 Naples, Italy; arturo.cesaro@unicampania.it; 2 Division of Cardiology, A.O.R.N. “Sant’Anna e San Sebastiano”, 81100 Caserta, Italy; 3 Department of Internal Medicine, School of Medicine, Federico II University, Via Sergio Pansini 5, 80131 Naples, Italy

## 1. Introduction

Cardiovascular diseases (CVDs) remain the leading cause of morbidity and mortality worldwide, with a growing impact on healthcare systems [1,2]. At the same time, cardiology is arguably the field of medicine in which progress is advancing most rapidly, with new diagnostic and therapeutic tools constantly reshaping clinical practice. Yet, important challenges remain. Heart failure (HF), atrial fibrillation (AF), pulmonary hypertension (PH), insulin resistance and hyperinsulinemia, and accelerated atherosclerosis in chronic inflammatory diseases are just a few examples of the broad spectrum of conditions that challenge clinicians on a daily basis [3,4,5,6,7]. At the same time, rapid progress in molecular biology, pharmacology, digital health, and artificial intelligence is opening new perspectives for earlier detection, improved prognostic stratification, and tailored therapeutic interventions [8,9,10].

This Special Issue, “Feature Reviews in Cardiovascular Diseases”, was conceived to provide an updated overview of these evolving fields, with a particular emphasis on mechanisms, biomarkers, and innovative therapies.

The six contributions published in this issue represent a cross-sectional view of the most promising directions in cardiovascular research and clinical practice, spanning from molecular mechanisms to real-world strategies.

## 2. Heart Failure: Biomarkers and Prognostic Factors

Wilk et al. [11] provide a comprehensive review of risk and prognostic markers in HF. Beyond established biomarkers such as natriuretic peptides and cardiac troponins, the authors highlight the emerging role of circulating microRNAs (miRNAs), including miR-30d, miR-126-3p, and miR-483-3p, as modulators of left ventricular remodeling, pulmonary pressure regulation, and right ventricular adaptation. The integration of novel imaging and monitoring tools—such as lung ultrasonography and remote dielectric sensing—further expands the capacity for personalized risk assessment. This work underscores the shift toward a multi-marker and multi-modality approach to HF management.

## 3. Super-Enhancer RNAs: New Molecular Targets

He et al. [12] review the intriguing field of super-enhancer RNAs (seRNAs), a novel class of non-coding RNAs transcribed from genomic super-enhancers. These molecules regulate critical genes involved in cell identity and disease progression, offering potential as diagnostic biomarkers and therapeutic targets. Although still at an early stage of investigation, strategies aimed at modulating seRNAs—via gene editing or pharmacological activators/inhibitors—represent an entirely new horizon for cardiovascular therapeutics. This article exemplifies how molecular discoveries can pave the way for precision medicine.

## 4. Early Detection of Atrial Fibrillation in Primary Care

The PREFATE study by Clua-Espuny and colleagues [13] addresses a crucial challenge: the early detection of AF in high-risk populations. Using mobile technology (Fibricheck^®^ app(CE certificate number BE16/819942412)) combined with echocardiography and ECG, the investigators identified new AF cases in more than 12% of screened individuals aged 65–85. Reduced left atrial strain and ejection fraction were strongly predictive of AF, but important evidence gaps were noted in guideline definitions of AF burden and anticoagulation thresholds. This prospective study demonstrates the feasibility of AF screening in primary care and highlights the urgent need for harmonized strategies to optimize stroke prevention.

## 5. Pulmonary Hypertension and Interstitial Lung Disease: An Expert Perspective

Criner et al. [14] offer an expert opinion on the complex interplay between pulmonary hypertension and interstitial lung disease (PH-ILD). While therapeutic options for pulmonary arterial hypertension and antifibrotic agents for ILD have expanded, their coexistence remains particularly challenging, associated with poor prognosis and increased mortality. The authors stress the importance of early recognition, comprehensive evaluation, and referral to specialized centers, including consideration for clinical trials and transplant. Their pragmatic, real-world perspective provides clinicians with practical insights into managing these high-risk patients.

## 6. Novel Cardiovascular Risk Biomarkers in Rheumatoid Arthritis

Pamies and colleagues [15] review the accelerated atherosclerotic burden of rheumatoid arthritis (RA), focusing on novel biomarkers that may enhance cardiovascular risk stratification. Interleukin-32, Dickkopf-1, galectin-3, catestatin, and fetuin-A emerge as promising candidates reflecting inflammation, endothelial dysfunction, fibrosis, and vascular calcification. While still investigational, these biomarkers have the potential to refine risk prediction and guide preventive strategies in RA, a condition with cardiovascular risk comparable to type 2 diabetes. This review reinforces the concept that systemic inflammation is a major driver of cardiovascular outcomes.

## 7. Artificial Intelligence and ECG: A New Frontier

The final contribution, by Bartusik-Aebisher and collaborators [16], examines the expanding role of artificial intelligence in ECG interpretation, particularly through wearable devices such as smartwatches. Deep learning algorithms are increasingly able to detect arrhythmias, heart failure, and ischemia, even from single-lead or photoplethysmography signals. While clinical validation and regulatory frameworks are still evolving, the integration of AI into digital cardiology holds promise for population-level screening and personalized preventive strategies. This article highlights the disruptive potential of digital health in reshaping cardiovascular diagnostics.

## 8. Integrative Perspectives

Taken together, the articles in this Special Issue illustrate the convergence of molecular innovation, digital technology, and clinical translation. From miRNAs and super-enhancer RNAs to AI-powered ECG analysis, from biomarker-driven risk prediction in RA to the complex management of PH-ILD, the contributions share a common theme: the need to identify disease earlier, stratify risk more precisely, and intervene in a more targeted and personalized manner.

The previous viewpoint by Fazio and Cesaro on insulin resistance/hyperinsulinemia reminds us that some of the most impactful cardiovascular risk factors remain insufficiently recognized [17]. A comprehensive approach to CVD must therefore integrate both cutting-edge discoveries and renewed attention to neglected but pervasive risk conditions.

This Special Issue provides an authoritative overview of the frontiers in cardiovascular research, bridging basic science, clinical practice, and digital innovation. Collectively, these works emphasize that the fight against cardiovascular disease requires both scientific foresight and multidisciplinary collaboration. Another key aspect emerging from this Special Issue is the need to overcome the traditional fragmentation between cardiovascular subspecialties and related disciplines. The evidence presented here, ranging from molecular biology to digital health applications, shows that the boundaries between basic research, clinical practice, and digital medicine are increasingly permeable. The cardiology of the future will inevitably be shaped by a close integration of translational biology, advanced imaging, artificial intelligence, and precision medicine, within a multidisciplinary framework that also involves pulmonologists, rheumatologists, internists, and primary care physicians. Only through such a shared vision can scientific innovation be effectively translated into strategies that reduce mortality and improve the quality of life of patients with cardiovascular disease.

As Guest Editors, we wish to thank all authors for their high-quality contributions, the reviewers for their constructive feedback, and the editorial staff of *Biomedicines* for their continuous support. We hope that this collection will serve not only as a reference point for current knowledge, but also as a catalyst for future research and innovation in cardiovascular medicine.

## References

[1] Vaduganathan M., Mensah G.A., Turco J.V., Fuster V., Roth G.A. (2022). The Global Burden of Cardiovascular Diseases and Risk. J. Am. Coll. Cardiol..

[2] Kazi D.S., Elkind M.S.V., Deutsch A., Dowd W.N., Heidenreich P., Khavjou O., Mark D., Mussolino M.E., Ovbiagele B., Patel S.S. (2024). Forecasting the Economic Burden of Cardiovascular Disease and Stroke in the United States Through 2050: A Presidential Advisory From the American Heart Association. Circulation.

[3] Cesaro A., Acerbo V., Scialla F., Scherillo G., De Michele G., Panico D., Porcelli G., de Sio V., Capolongo A., Sperlongano S. (2025). Role of LipoprotEin(a) in CardiovascuLar Diseases and Premature Acute Coronary Syndromes (RELACS Study): Impact of Lipoprotein(a) Levels on the Premature Coronary Event and the Severity of Coronary Artery Disease. Nutr. Metab. Cardiovasc. Dis..

[4] Van Gelder I.C., Rienstra M., Bunting K.V., Casado-Arroyo R., Caso V., Crijns H.J.G.M., De Potter T.J.R., Dwight J., Guasti L., Hanke T. (2024). 2024 ESC Guidelines for the Management of Atrial Fibrillation Developed in Collaboration with the European Association for Cardio-Thoracic Surgery (EACTS). Eur. Heart J..

[5] Mach F., Koskinas K.C., Roeters van Lennep J.E., Tokgözoğlu L., Badimon L., Baigent C., Benn M., Binder  C.J., Catapano A.L., De Backer  G.G. (2025). ESC/EAS Scientific Document Group. 2025 Focused Update of the 2019 ESC/EAS Guidelines for the management of dyslipidaemias. Atherosclerosis.

[6] Marx N., Federici M., Schütt K., Müller-Wieland D., Ajjan R.A., Antunes M.J., Christodorescu R.M., Crawford C., Di Angelantonio E., Eliasson B. (2023). 2023 ESC Guidelines for the Management of Cardiovascular Disease in Patients with Diabetes. Eur. Heart J..

[7] McDonagh T.A., Metra M., Adamo M., Gardner R.S., Baumbach A., Böhm M., Burri H., Butler J., Čelutkienė J., Chioncel O. (2023). 2023 Focused Update of the 2021 ESC Guidelines for the Diagnosis and Treatment of Acute and Chronic Heart Failure. Eur. Heart J..

[8] Cesaro A., Pastori D., Acerbo V., Biccirè F.G., Golino M., Panico D., Prati F., Abbate A., Lip G.Y.H., Calabrò P. (2025). Reduction of New Onset of Atrial Fibrillation in Patients Treated with Semaglutide: An Updated Systematic Review and Meta Regression Analysis of Randomized Controlled Trials. Eur. J. Prev. Cardiol..

[9] Costa F., Gomez Doblas J.J., Díaz Expósito A., Adamo M., D’Ascenzo F., Kołtowski L., Saba L., Mendieta G., Gragnano F., Calabrò P. (2025). Artificial Intelligence in Cardiovascular Pharmacotherapy: Applications and Perspectives. Eur. Heart J..

[10] Tamargo J., Agewall S., Borghi C., Ceconi C., Cerbai E., Dan G.A., Ferdinandy P., Grove E.L., Rocca B., Magavern E. (2024). New Pharmacological Agents and Novel Cardiovascular Pharmacotherapy Strategies in 2023. Eur. Heart J. Cardiovasc. Pharmacother..

[11] Wilk M.M., Wilk J., Urban S., Gajewski P. (2024). Current Review of Heart Failure-Related Risk and Prognostic Factors. Biomedicines.

[12] He Y., Cai Y., Cao Y., Wang Y., Wang J., Ding H. (2025). Application Strategies of Super-Enhancer RNA in Cardiovascular Diseases. Biomedicines.

[13] Clua-Espuny J.L., Hernández-Pinilla A., Gentille-Lorente D., Muria-Subirats E., Forcadell-Arenas T., de Diego-Cabanes C., Ribas-Seguí D., Diaz-Vilarasau A., Molins-Rojas C., Palleja-Millan M. (2025). Evidence Gaps and Lessons in the Early Detection of Atrial Fibrillation: A Prospective Study in a Primary Care Setting (PREFATE Study). Biomedicines.

[14] Criner R.N., Naranjo M., D’Alonzo G., Weaver S. (2025). Pulmonary Hypertension-Related Interstitial Lung Disease: An Expert Opinion with a Real-World Approach. Biomedicines.

[15] Pamies A., Vallvé J.-C., Paredes S. (2025). New Cardiovascular Risk Biomarkers in Rheumatoid Arthritis: Implications and Clinical Utility—A Narrative Review. Biomedicines.

[16] Bartusik-Aebisher D., Rogóż K., Aebisher D. (2025). Artificial Intelligence and ECG: A New Frontier in Cardiac Diagnostics and Prevention. Biomedicines.

[17] Fazio S., Affuso F., Cesaro A., Tibullo L., Fazio V., Calabrò P. (2024). Insulin Resistance/Hyperinsulinemia as an Independent Risk Factor That Has Been Overlooked for Too Long. Biomedicines.

